# High-throughput screening for respiratory pathogens within pigs in Denmark; analysis of circulating porcine respiratory coronaviruses and their association with other pathogens

**DOI:** 10.1016/j.virusres.2024.199501

**Published:** 2024-11-26

**Authors:** Amalie Ehlers Bedsted, Nicole B. Goecke, Charlotte K. Hjulsager, Pia Ryt-Hansen, Kasama Chusang Larsen, Thomas Bruun Rasmussen, Anette Bøtner, Lars E. Larsen, Graham J. Belsham

**Affiliations:** a Department of Veterinary and Animal Sciences, Faculty of Health and Medical Sciences, University of Copenhagen, Dyrlægevej 88 1870 Frederiksberg, Denmark; b Department of Virus and Microbiological Special Diagnostics, Statens Serum Institut, Artillerivej 5 2300 Copenhagen, Denmark; c Center for Diagnostics, Department of Health Technology, Technical University of Denmark, Henrik Dams Allé 202 2800 Kgs. Lyngby, Denmark

**Keywords:** Porcine respiratory disease complex (PRDC), Porcine respiratory coronavirus (PRCV), Swine influenza a virus (swIAV), Respiratory bacterial pathogens, Coronaviruses

## Abstract

Porcine respiratory coronavirus (PRCV) typically causes subclinical or mild respiratory infections in pigs, but may lead to more severe disease with other factors. PRCV infection in Denmark was initially detected in 1984, but data are lacking about its current prevalence and diversity. Antibodies against PRCV were detected in about 75 % of recent pig sera from Denmark. In addition, pig nasal swab samples were screened for PRCV and 12 other respiratory pathogens using a high-throughput RT-qPCR system. All targeted pathogens were detected but at different prevalences. Significant associations were found between the presence of PRCV and certain other pathogens. From PRCV positive samples, partial spike gene sequences and complete nucleocapsid coding sequences were determined. In phylogenetic analyses, these PRCVs clustered with earlier European PRCVs and were distinct from transmissible gastroenteritis virus. We conclude that PRCV is widespread within the pig population in Denmark. Further studies on the significance of PRCV are warranted.

## Highlights

• Serological and RT-qPCR analyses showed that PRCV is present in pigs in Denmark.
• Associations observed between the presence of PRCV and other respiratory pathogens.
• Sequences were obtained for the complete PRCV N gene from nasal swabs.
• Sequences were obtained for part of the PRCV S gene from nasal swabs.
• In phylogenetic analyses, these sequences clustered with earlier European strains.

## 1 Introduction

Respiratory disease is one of the major health concerns in pigs (Kongsted and Sørensen, 2017). Multiple different pathogens can lead to respiratory disease in pigs, including viruses and bacteria (Brockmeier et al., 2002). Collectively, this disease is known as the porcine respiratory disease complex (PRDC) (Brockmeier et al., 2002). In 2022, the pig population in Denmark consisted of about 12 million animals (in October) on approximately 2400 farms, and over 30 million pigs were produced in that year (Danish Agriculture and Food Council, 2023). In Denmark, one of the major pathogens causing respiratory disease in pigs is swine influenza A virus (swIAV). From passive surveillance, based on submitted samples from pigs with clinical signs consistent with swIAV infection (SSI, 2024), the prevalence of swIAV in Denmark in 2023 was 61.3 % (293/478 submissions) (SSI, 2024).

Another virus that may also contribute to the development of PRDC is porcine respiratory coronavirus (PRCV) (Brockmeier et al., 2002), which is a deletion variant of transmissible gastroenteritis virus (TGEV) (Rasschaert et al., 1990). PRCV and TGEV are members of the virus subfamily *Orthocoronavirinae*, which are enveloped, positive sense, single stranded RNA viruses with genome sizes ranging from 26 to 31 kb (Woo et al., 2023). PRCV and TGEV both belong to the genus *Alphacoronavirus* and have four structural proteins, these are the spike (S), envelope (E), membrane (M), and nucleocapsid (N) proteins (Woo et al., 2023). The deletion in the PRCV genome, compared to the TGEV sequence, is located within the S gene; the deletion varies in size between 621 and 681 nucleotides (nt) and it can be used to differentiate between TGEVs and PRCVs (Bedsted et al., 2024; Paul et al., 1997; Rasschaert et al., 1990). TGEV, a notifiable agent in Denmark, has never been detected in the country (Danish Agriculture and Food Council, 2013). Using samples collected in 1985–86, it was found that pigs in 58.8 % of the Danish herds had antibodies against PRCV (Henningsen et al., 1988) and a seropositivity rate of 75–80 % in pigs in Denmark was described by Have (1990). In 1990, a PRCV strain was isolated in Denmark from a pig nasal swab sample, and the complete genome sequence of this virus (1/90-DK) was recently determined (Bedsted et al., 2024). However, studies on the current prevalence and nature of PRCV in pigs in Denmark are lacking.

In 2020, a screening assay based on reverse transcription followed by use of a high-throughput quantitative real-time polymerase chain reaction (RT-qPCR) system for the detection of respiratory and enteric pathogens in pigs was developed. This system proved to be highly specific and sensitive (Goecke et al., 2020a). Using this method, it was shown that a wide range of different respiratory pathogens were circulating in pigs in Denmark, however, that study did not include PRCV (Goecke et al., 2020b). In the present study, the same screening method was used to determine the prevalence of 13 different respiratory pathogens, including PRCV (see Materials and Methods for details of the agents screened for), in Denmark using samples collected from pigs during the period 2021 to 2023.

In a recent study from Spain and Portugal, it was found that (at a farm-level), PRCV, swine orthopneumovirus (SOV), and porcine cytomegalovirus (PCMV) were more likely to be found in swIAV positive, compared to negative, nurseries (Martín-Valls et al., 2022).

The aims of the present study were, therefore, to investigate the presence of a collection of different respiratory pathogens, including PRCV, in samples from pigs from Denmark. Furthermore, in order to characterize the viruses in samples that tested positive for PRCV, we sequenced the complete N gene and a part of the S gene (831 nt) from some of the positive samples and compared them to the previously published PRCV genome sequences.

## 2 Materials and methods

### 2.1 Serology

For the analysis of anti-PRCV antibodies in young female pigs (gilts) from Denmark, between 8 and 30 samples from each of 21 sow herds were obtained, in total 255 samples. The samples were collected in a project that was described previously in a report (Nielsen et al., 2023), although the herd numbers used in the present study and in the report are not the same. The samples were collected in 2021 and 2022 from gilts of approximately five to nine months of age. The gilts were bought from a supplier or were recruited internally and in most cases placed in quarantine before entering the sow herds, and the samples were collected at the time of exit from the quarantine. All herds bought gilts from the same supplier, except for two herds (herds 8 and 17) that reared their own pigs (Nielsen et al., 2023). The quarantine period varied between 6 and 14 weeks, and the quarantine conditions also varied. Some herds used an “all-in-all-out” principle in the quarantine stable, and others had a continuous intake of gilts (Nielsen et al., 2023). Four herds (herds 3, 6, 8, and 17) did not use quarantine before the gilts were introduced into the sow herds.

Anti-PRCV antibodies in serum samples were detected using the Swinecheck® TGEV/PRCV Recombinant kit (Biovet Inc., QC, Canada), and samples with inhibition percentages of 40 % or above were considered positive. The kit was used as described by the manufacturer with the exception that only the conjugate detecting anti-PRCV antibodies was used. The assay can also detect TGEV, but since TGEV has never been detected in Denmark, we will refer to these antibodies as anti-PRCV antibodies. All samples were run in duplicate. One sample (from herd number 3) was excluded due to a high degree of hemolysis, and the total number of samples (255) excludes this single sample.

### 2.2 Sampling groups assayed by RT-qPCR

For the detection of respiratory pathogens in samples from pigs in Denmark, three different groups of samples were tested (Table 1). All the samples had originally been obtained for diagnostic (groups 1 and 3) or research (group 2) purposes.

**Table 1:** Analysis of nasal swab samples collected from pigs in Denmark. The numbers of nasal swab samples in each group, swIAV RT-qPCR status, and sampling period are shown. The swIAV results were obtained using three different assays, swIAV (Nagy2) (group 1) (supplementary materials, Table S1), an RT-qPCR assay described previously (group 2) (Kristensen et al., 2023), and swIAV (Inf M) (group 3) (supplementary materials, Table S1). The samples in the groups 1, 2, and 3 consisted of pooled nasal swabs.

Group number	swIAV pos. samples	swIAV neg. samples	Total number of samples (pools)	Number of sampled pigs per pool	Collection period
1	90	86	176	5 (average)	December 2022 to October 2023
2	0	30	30	3–5	July 2021 to January 2022
3	37	88	125	10	May to July 2022
All groups	127	204	331	3–10	July 2021 to October 2023

Group 1 contained 176 pooled nasal swabs from 143 submissions (a single submission may contain multiple pooled swab samples). Each pool consisted of nasal swab samples from five pigs on average. The samples were collected within the period from December 2022 to October 2023 from pigs with respiratory symptoms. Of these samples, a specific collection date had not been registered for 10/176 samples, but submission dates were available, and these samples were obtained during the same period as the others. After testing with an RT-qPCR assay (Nagy2, supplementary materials, Table S1) targeting the matrix (M) gene of swIAV, the samples were divided into two groups, swIAV positive (90 samples), and swIAV negative (86 samples). The ages of the sampled pigs were registered for approximately 2/3rds of the pooled samples and varied from 1 to 12 weeks of age, except for one older pig that was between 11 and 20 weeks of age.

Group 2 consisted of 30 pooled nasal swab samples from eight PRCV seropositive herds out of the 21 tested (see above). The samples were collected between July 2021 and January 2022. Each pool contained nasal swab samples from three to five gilts, and in total, samples from 143 pigs were tested. All the samples had been tested negative for swIAV with an RT-qPCR assay targeting the M gene, as described previously (Kristensen et al., 2023). No clinical information is available for this group, and the ages of the gilts ranged from approximately five to nine months of age. The groups of gilts that were tested for serum antibodies by ELISA and viral RNA levels in nasal swabs were overlapping, but not identical. Therefore, and due to the fact that the nasal swab samples were pooled, and the serum samples were not, individual serum antibody levels and viral RNA levels in the nasal swab samples could not be compared.

Group 3 consisted of 125 pooled nasal swab samples collected from pigs during May to July 2022. These samples were pooled with 10 swabs per pool. For 38 of the 125 samples, no specific date of collection was registered, but these samples were obtained during the same period as the others. The samples were collected as part of an active surveillance program, and were mostly collected from pigs with a variety of symptoms such as diarrhea and coughing. The ages of these pigs were not registered. Using an RT-qPCR assay targeting the M gene (Inf M assay, supplementary materials Table S1), 37 of the samples tested positive and 88 tested negative for swIAV.

### 2.3 RNA and DNA extractions

Nucleic acids were extracted from the pooled nasal swab samples from group 1 using a MagNAPure 96 extraction robot (Roche, Basel, Switzerland) with the MagNA Pure 96 DNA and Viral NA Small Volume Kit (Roche Diagnostics, Copenhagen, Denmark). From the nasal swab samples from the groups 2 and 3, nucleic acids were extracted on a Qiacube HT extraction robot (Qiagen, Hilden, Germany), using the IndiSpin QIAcube HT Pathogen Kit (Indical Bioscience, Leipzig, Germany).

### 2.4 Primers and probes for detection of respiratory pathogens by RT-qPCR

We screened for various pathogens by RT-qPCR using primers and probes described previously for *Actinobacillus pleuropneumoniae, Bordetella bronchiseptica,* swIAV (Inf M), *Mycoplasma hyopneumoniae, Mycoplasma hyorhinis*, PCMV, PCV2, *Stretococcus suis* type 2 (Goecke et al., 2020a, 2020b), swIAV (Nagy2) (Nagy et al., 2021), PRCV (Keep) (Keep et al., 2022), PRCV (Kim) (Kim et al., 2007), PRCV (MV) (Martín-Valls et al., 2022), porcine respirovirus (PRV)1 and SOV (Graaf-Rau et al., 2023); some were slightly modified (supplementary materials, Table S1). The primers, but not the probe, targeting the porcine housekeeping gene *PPIA* (peptidylprolyl isomerase A), used as a positive internal control, have been published previously (Starbæk et al., 2022).

Primers and probes for the detection of *Pasteurella multocida* and *Glaesserella. (formerly Haemophilus) parasuis* were designed based on the sequences of the genes encoding the histone-lysine N-methyltransferase 1 (Kmt1) protein and the outer membrane protein P2 (OmpP2), respectively, following alignment of multiple sequences from GenBank (National Center for Biotechnology Information (NCBI), 2024) (supplementary materials, Table S1). The properties of the primers and probes were characterized utilizing the OligoAnalyzer™ Tool (IDT). Additionally, the primer specificity was tested *in silico* using the Primer-BLAST tool (National Center for Biotechnology Information (NCBI), 2024). Primer and probe sensitivity, specificity, and amplification efficiency were assessed, in duplicate, using a 10-fold serial dilution of positive control samples.

### 2.5 Reverse transcription and pre-amplification for high-throughput qPCR

Following nucleic acid extraction, all samples were processed using the High-Capacity cDNA Reverse Transcription Kit (Applied Biosystems, Thermo Fisher Scientific, Denmark), according to the kit manual with the exception that a total volume of 10 µl (rather than 20 µl) was used per sample. After cDNA synthesis, the TaqMan preAmp Master Mix kit (Applied Biosystems) was used for pre-amplification as described previously (Goecke et al., 2020a). Both the cDNA synthesis and the pre-amplification described above were run on a PCRmax thermocycler (Cole-Parmer (Antylia Scientific), Vernon Hills, IL, USA).

### 2.6 High-throughput qPCR

A 192.24 dynamic array (DA) integrated fluidic circuit (IFC) chip (Standard BioTools, San Francisco, CA, USA) was loaded with samples and assay mixes, and then placed in an IFC controller RX (Standard BioTools) for final loading and mixing as described previously (Goecke et al., 2021). The high-throughput qPCR system BioMark HD (Standard BioTools) was used essentially as described by Goecke et al. (2021). Some assays were run in duplicate (for *B. bronchiseptica, G. parasuis,* PRV1, and PRCV), and the remaining assays as single reactions. Positive (extracted and pre-amplified nucleic acids from samples known to be positive for each of the pathogens) and negative controls (with water instead of nucleic acids) were included in each run. The data were analyzed according to cycle threshold (Ct) value and amplification curves using the Standard BioTools Real-Time PCR analysis (version 1.0.2) software.

In general, the pre-amplified samples were considered positive if they had a Ct below 25. For the swIAV (Inf M) assay and the *A. pleuropneumoniae* assay, Ct values up to 27 were considered positive. Samples that yielded odd-looking amplification curves were regarded as negative. If the samples were run in duplicate assays, i.e. using the same test, they had to be scored positive in both assays to be considered positive. If the samples had been run twice, they were considered positive if they were both positive or if one sample had a convincing amplification curve and a Ct below 21.

For the PRCV assays, four out of the six assay replicates had to be positive for the sample to be scored positive. For the group 1 samples, one of the PRCV assay (PRCV (Kim)) replicates did not run well, and so the results were excluded. Thus, for these samples, a sample was considered positive if at least four out of five assays were positive.

The group 3 samples had been tested previously by high-throughput RT-qPCR for 10 of the pathogens detected in our study, and these results were compared to those obtained in this study. Following this comparison, one sample tested with the swIAV (Inf M) assay (supplementary materials, Table S1) that had previously been considered positive (with a Ct of 20.9), was scored as negative.

### 2.7 Associations between pathogens

Only samples from group 1 were included in the analysis for potential associations between the detections of the included pathogens. The high-throughput RT-qPCR results were transformed into categorical, dichotomous data (positive/negative), depending on the amplification curve and the Ct value as described above. In order to analyze whether there was an association between samples positive for swIAV by RT-qPCR, as determined using the swIAV (Nagy2) assay, and for other pathogens as indicated above, the results were analyzed in a χ^2^ test, or a Fisher's exact test. The Fisher's exact test was used if at least one value in the 2 × 2 table was equal to, or below, five. For PRCV, only the final number of positive samples was included (4/5 replicates, as described above). Associations were considered statistically significant if *P* < 0.05. Furthermore, a risk ratio (RR) and a 95 % confidence interval (CI) for each combination was calculated. The 95 % CI was calculated using the Koopman asymptotic score method (Koopman, 1984). Associations between PRCV and other pathogens were also analyzed with χ^2^ tests, or Fisher's exact tests, and RR calculations, as above. The statistical tests χ^2^, Fisher's exact, and RR calculations were performed using GraphPad Prism 10.2.2.

### 2.8 Assessment of seasonal variation in the proportion of PRCV positive samples

The samples from pigs in group 1 were analyzed to see whether the percentage of PRCV positive samples varied across the year. As above, only the final number of positive samples was included (4/5 replicates). Only samples with a registered date of collection were included in this analysis, in total 166. The percentage of PRCV positive samples was calculated for each month. Samples from the other groups were not included since either these did not have any PRCV positive samples (group 2) or had a shorter time period of collection (group 3).

### 2.9 PRCV N gene and partial S gene sequencing

Some of the samples in group 1 with the lowest Ct values in the PRCV assays (i.e. highest levels of PRCV RNA) were selected for further analysis. Initially, the N gene and a part of the S gene were amplified by conventional RT-PCR and the amplicons were analyzed by Sanger sequencing. The part of the S gene chosen spanned 831 nt, outside of the region deleted in PRCV, and included the coding sequence for nine amino acid residues, which had been identified as specific for either PRCVs from Europe or from the US by Bedsted et al., 2024. The whole N gene open reading frame (1149 nt) was sequenced. The cDNA was synthesized using the SuperScript™ III first-strand system kit (Invitrogen, Thermo Fisher Scientific, Denmark) from the viral RNA extracted as above. Synthesis of cDNA was performed according to the kit manual, with the exception that we used a mixture of primers (up to six different) including the PRCV specific primers 22283R, 22437R, PRCVseqSR, and PRCVseqNR2 (supplementary materials, Table S2), as well as the unspecific primers oligo(dT)_20_ and random hexamers (pN6) from the kit. The cDNA was amplified using conventional PCRs (GeneAmp PCR system 9700, Applied Biosystems, Waltham, MA, USA) with the primer combinations: PRCVseqSF and 22437R or PRCVseqSR for S, and PRCVseqNF1 and PRCVseqNR2 for N (supplementary materials, Table S2). For the PCR, either the AccuPrime™ *Pfx* DNA Polymerase system, high fidelity (Invitrogen) was used or the Phusion Hot Start II Hi-Fi DNA Polymerase system (Thermo Scientific, Thermo Fisher Scientific, Denmark); both assays were run according to the kit manuals. When an amplicon was observed after gel electrophoresis, the DNA fragment was excised from the gel and purified using the GeneJET Gel Extraction Kit (Thermo Scientific). The PCRs and gel extractions were performed according to the manufacturer's manuals. The amplicons were Sanger sequenced (LGS Genomics GmbH, Berlin, Germany) in both forward and reverse directions using the same primers that had been used for the conventional PCR and cDNA synthesis and with internal primers when required to complete the sequence (supplementary materials, Table S2).

In some cases, for both the S gene and the N gene sequences, we obtained sequences with ambiguous nt in some positions. For some S gene sequences, this was resolved by inserting the gel purified PCR products into TOPO vectors, followed by transformation into competent *E. coli* cells. The plasmids were isolated from the resulting colonies, after growth in liquid culture, using the GeneJET Plasmid Miniprep kit (Thermo Scientific). Finally, the inserts in the plasmids were Sanger sequenced (LGS), and consensus sequences were identified.

### 2.10 Construction of phylogenetic trees based on the PRCV N gene and partial S gene sequences

The N gene (1149 nt) and partial S gene (831 nt) sequences were compared in separate alignments using MAFFT v7.490 along with 47 TGEV sequences, and 13 (N) or 14 (S) PRCV sequences from either Europe or the US that were downloaded from GenBank in February 2024 (National Center for Biotechnology Information (NCBI), 2024). For TGEV, available complete genome sequences were downloaded, and for PRCV, full S and/or N gene sequences from Europe and the US were downloaded. The list of TGEV and PRCV sequences used can be found in the supplementary materials, Table S3. Based on these alignments, phylogenetic trees were built with MrBayes 3.2.6 as described by Bedsted et al., 2024 using Geneious Prime 2024.0.2.

### 2.11 Graphs

All graphs were prepared using GraphPad Prism 10.2.2.

## 3 Results

### 3.1 Detection of anti-PRCV antibodies in pig sera

In total, anti-PRCV antibodies were detected in 191 out of the 255 serum samples (74.9 %) tested, which were from 19 out of the 21 herds (90.5 %) (Table 2), i.e. no samples from two herds (herds 4 and 20) had detectable anti-PRCV antibodies. In contrast, all samples (100 %) tested from 11 of the 21 herds (52.3 %) had such antibodies.

**Table 2:** Prevalence of anti-PRCV antibodies in Danish pig herds.

Herd no.	Number of positive samples	Number of samples tested	Prevalence (%)
1	26	30	86.7
2	1	8	12.5
3	8	8	100
4	0	19	0
5	10	19	52.6
6	14	16	87.5
7	11	18	61.1
8	15	18	83.3
9	8	8	100
10	15	15	100
11	8	9	88.9
12	8	10	80.0
13	9	9	100
14	8	8	100
15	8	8	100
16	10	10	100
17	8	8	100
18	8	8	100
19	8	8	100
20	0	10	0
21	8	8	100

**Fig. 1:**
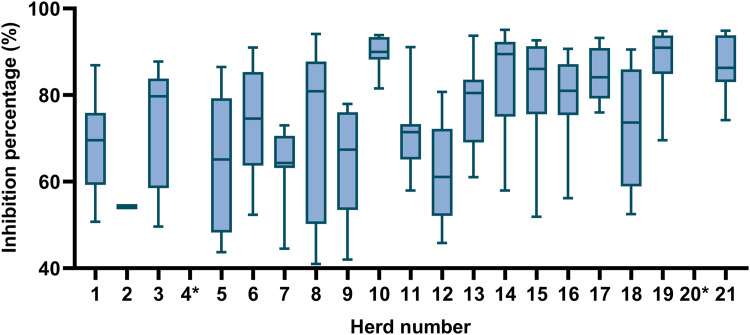
Range of inhibition percentage values observed for pig serum samples with anti-PRCV antibodies from different herds. Maximum, mean, and minimum value of the inhibition percentage for positive samples. Threshold value for positive samples: ≥40 %. * Herds 4 and 20 did not have any positive samples.

In general, there was a large variation in the inhibition percentage observed in the assay among the positive samples, corresponding to different levels of seropositivity. However, the samples from herd 10 all gave relatively high inhibition percentage values (Fig. 1).

### 3.2 Detection of PRCV and other respiratory pathogens by high-throughput RT-qPCR

Each of the 13 pathogens that were tested for, were detected in at least some of the pig swab samples. Across all sample groups, most nasal swab samples contained detectable levels of *G. parasuis* (318/331; 96.1 %) and *S. suis* type 2 (330/331; 99.7 %). The least prevalent pathogen was PCV2 (26/331; 7.9 %) (Table 3).

**Table 3:** Heat map for the screening results of pig nasal swab samples for 13 different pathogens by RT-qPCR. Number (n) and frequency (%) of positive nasal swab samples in the groups 1, 2, and 3 (as described in Table 1 and in Materials and Methods). The heat map colors represent three different frequency intervals: White 0–29.9 %, yellow 30–69.9 %, red 70–100 %. Abbreviations for the pathogens are as follows: A. pleuropneumoniae (AP), B. bronchiseptica (BB), G. (formerly Haemophilus) parasuis (HP), swine influenza A virus (swIAV), M. hyopneumoniae (M. hyop.), M. hyorhinis (M. hyorhi.), porcine cytomegalovirus (PCMV), porcine circovirus 2 (PCV2), P. multocida (PM), porcine respiratory coronavirus (PRCV), porcine respirovirus 1 (PRV1), swine orthopneumovirus (SOV), S. suis type 2 (SS).

^1^These results differ from what was determined initially in separate assays (Table 1), however, they are still within the same heat map categories.

From Table 3, it is apparent that the two different swIAV assays and the three PRCV assays do not agree completely in terms of the number of positive and negative samples, although they were very similar. Furthermore, it should be noted that the swIAV results in Table 3 are not identical to those obtained previously for groups 1 (using the Nagy2 assay) and 3 (using the Inf M assay) (Table 1).

### 3.3 Associations between pathogens

**Fig. 2:**
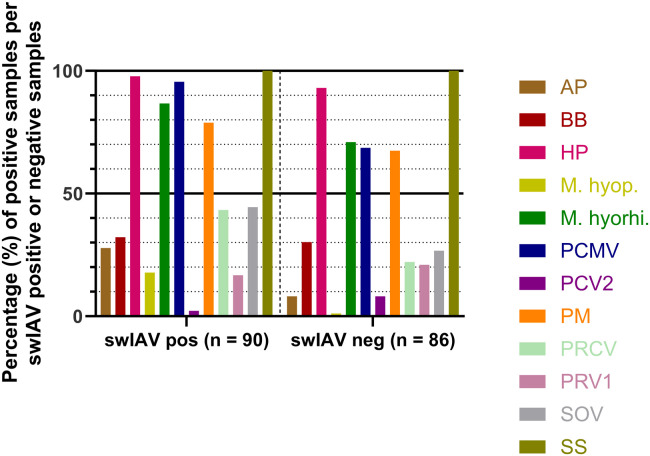
Percentage of respiratory pathogens in samples from pigs with or without swIAV. Positive nasal swab samples, calculated as the percentages of samples that were positive for each pathogen for the swIAV positive and negative samples (determined using the swIAV (Nagy2) assay), respectively. Abbreviations as in Table 3.

Fig. 2 shows the distribution of the nasal swab samples from group 1 that tested positive for the 12 other pathogens grouped by swIAV positive and negative status. In the swIAV negative samples, the most common pathogens were *S. suis* type 2 (100 %; 86/86), *G. parasuis* (93.0 %; 80/86), *M. hyorhinis* (70.9 %; 61/86), PCMV (68.6 %; 59/86), and *P. multocida* (67.4 %; 58/86). All of these pathogens were also highly prevalent in the swIAV positive samples (Fig. 2). Two pathogens, PCV2 and PRV1, appeared more prevalent in swIAV negative samples (PCV2: 8.1 %; 7/86, PRV1: 20.9 %; 18/86) than in swIAV positive samples (PCV2: 2.2 %; 2/90, PRV1: 16.7 %; 15/90) (Fig. 2).

Using χ^2^ or Fisher's exact tests, there were found to be statistically significant associations (*P* < 0.05) between the presence of swIAV and the presence of *A. pleuropneumoniae, M. hyopneumoniae, M. hyorhinis*, PCMV, PRCV, and SOV in the group 1 samples (Table 4). The presence of swIAV significantly increased the risk of the presence of these pathogens (Table 4). In contrast, there was no apparent association between the presence of swIAV and certain other pathogens, e.g. *B. bronchiseptica* and PRV1 (Table 4). All samples were positive for *S. suis* type 2, and so this pathogen was excluded from this analysis, and from that for PRCV (Table 4, Table 5).

**Table 4:** Associations between the presence of swIAV and the presence of other pathogens in nasal swab samples. Only samples from group 1 were included. As all samples were positive for S. suis type 2, this pathogen was not included in the analysis. The test performed was either a χ^2^ or Fisher's exact (FE) test as indicated, and the relative risk (RR) was calculated.

Pathogen	Test	*P*	RR	95 % CI for RR
*A. pleuropneumoniae*	χ^2^	<0.001*	1.73	[1.29, 2.21]
*B. bronchiseptica*	χ^2^	0.78	1.05	[0.76, 1.40]
*G. parasuis*	FE	0.16	2.10	[0.87, 7.38]
*M. hyopneumoniae*	FE	<0.001*	2.02	[1.52, 2.46]
*M. hyorhinis*	χ^2^	0.01*	1.73	[1.12, 2.91]
PCMV	FE	<0.001*	4.60	[2.03, 11.65]
PCV2	FE	0.09	0.42	[0.12, 1.06]
*P. multocida*	χ^2^	0.09	1.36	[0.96, 2.05]
PRCV	χ^2^	0.003*	1.56	[1.17, 2.04]
PRV1	χ^2^	0.47	0.87	[0.56, 1.24]
SOV	χ^2^	0.01*	1.44	[1.08, 1.90]

⁎ Significant results (*P* < 0.05).

**Table 5:** Associations between the presence of PRCV and the presence of other pathogens in nasal swab samples. Only samples from group 1 were included. As all samples were positive for S. suis type 2, this agent was not included in the analysis. The test performed was either a χ^2^ or Fisher's exact (FE) test, and for each pathogen, the relative risk (RR) was calculated.

Pathogen	Test	*P*	RR	95 % CI for RR
swIAV	χ^2^	0.003*	1.96	[1.25, 3.13]
*A. pleuropneumoniae*	χ^2^	0.55	1.17	[0.68, 1.86]
*B. bronchiseptica*	χ^2^	0.70	0.91	[0.56, 1.42]
*G. parasuis*	FE	0.054	−^1^	−^1^
*M. hyopneumoniae*	χ^2^	0.06	1.72	[0.96, 2.64]
*M. hyorhinis*	FE	0.005*	2.82	[1.32, 6.60]
PCMV	FE	0.003*	3.92	[1.48, 11.52]
PCV2	FE	0.27	0.33	[0.06, 1.31]
*P. multocida*	χ^2^	0.047*	1.75	[1.01, 3.22]
PRV1	χ^2^	0.44	0.80	[0.42, 1.37]
SOV	χ^2^	<0.001*	2.06	[1.36, 3.11]

^1^Since there was a 0 in the contingency table, the RR and 95 % for RR could not be calculated.

CI = “confidence interval”.

⁎ Significant results (*P* < 0.05).

Furthermore, using the same method, there were statistically significant associations (*P* < 0.05) found between the presence of PRCV and the presence of *M. hyorhinis,* PCMV, *P. multocida,* and SOV in the group 1 samples. The presence of PRCV increased the risk of the presence of these pathogens (Table 5).

### 3.4 Assessment of seasonal variation in the proportion of PRCV positive samples

**Fig. 3:**
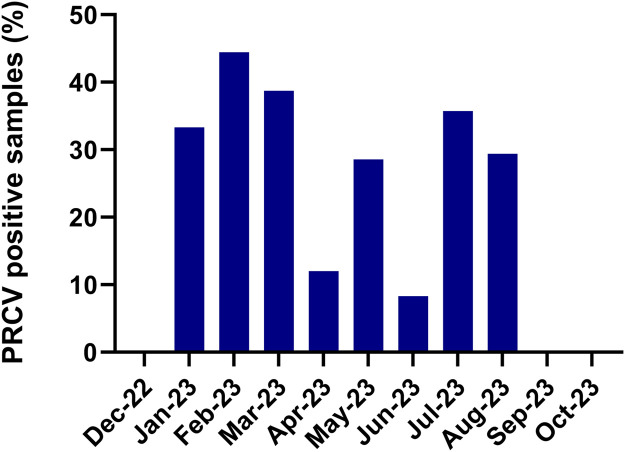
Proportion of PRCV positive nasal swab samples (%) in the months between December 2022 and October 2023. The total number of included samples was 166.

Based on the samples from group 1, there was no apparent seasonal pattern in the detection of PRCV from samples collected from December 2022 to October 2023 (Fig. 3); the percentage of PRCV positive samples was approximately as high in July (35.7 %; 5/14) and August (29.4 %; 5/17) 2023, as in January (33.3 %; 8/24), February (44.4 %; 8/18), and March (38.7 %; 12/31) 2023. The average number of tested samples per month was 15, however, only one, three, and seven of the tested samples were from December 2022, September, and October 2023, respectively, and all samples from these months were negative for PRCV in the screening (Fig. 3).

### 3.5 PRCV N gene and partial S gene sequencing

In total, we obtained 18 partial S gene (831 nt) sequences from 16 samples, and 20 complete N gene open reading frame sequences (1149 nt) from 20 samples and both the partial S gene and the N gene sequences from 14 samples. The sequence names were assigned as PRCV/DK/23/1–22 followed by N or S, respectively, and GenBank accession numbers are indicated in the supplementary materials, Table S4. For the S gene sequences, two different consensus sequences were obtained from the samples giving the sequences PRCV/DK/23/14 S (14.1 and 14.2 S) and PRCV/DK/23/21 S (21.1 and 21.2 S). However, for the N gene sequences, three sequences remained ambiguous (PRCV/DK/23/11, 16, and 18 N) with one ambiguous nt, which did not lead to an amino acid change.

At the nt level, the S gene sequences from these pig nasal swabs were 97.5–99.9 % identical to each other and 95.5–96.4 % identical to the 1/90-DK sequence (acc. no. OK078898.1) isolated in Denmark over 30 years previously. The N gene sequences were 96.2–99.8 % identical to each other, and 95.6–96.7 % identical to the 1/90-DK strain at the nt level.

For the predicted S protein amino acid sequences, out of the nine encoded amino acid residues that were identified as being unique for the published European PRCV sequences (Bedsted et al., 2024), seven were also present in the new Danish sequences from the nasal swab samples (note: the amino acid residue numbers are based on the sequence of the PRCV LEPP strain (acc. no. OR209252.1)). However, two residues (V538 and I579) were the same as those found in all the US strains, except for the PRCV/DK/23/21.1 S sequence that encoded residue L579 uniquely among all of these PRCV sequences. Following a BLAST search with the 831 nt long sequence, it was apparent that the presence of the encoded residue L579 was unique among the 100 most similar PRCV and TGEV sequences (National Center for Biotechnology Information (NCBI), 2024). The amino acid residues of strain RM4 in these positions were identical to those of the other European strains, while the HOL87 strain varied at one position (residue V538) (Table 6).

**Table 6:** Predicted amino acid residues in the PRCV S protein at positions that distinguish US and European strains. The accession numbers are as follows for the seven US strains: DQ811787.1, KR270796.1, KY406735.1, OM830319.1, OR209252.1, OR209253.1, and OR209254.1, and for the five European strains: OK078898.1, OR689863.1, OR689864.1, OM830318.1, and OM830320.1. The residue numbering is based on the sequence OR209252.1 (LEPP). X/Y indicates that two alternative amino acids can be found at this position. Amino acids predicted in the newly sequenced strains from Denmark, or in RM4 (from 1986) and HOL87 (from 1987) that are different from the other European PRCVs are highlighted in green. Underlined amino acids are unique at this position.

The newly sequenced PRCV strains (from the period 2022–23) from Denmark and the HOL87 (from 1987) sequence encode residue V538 as in the US PRCV sequences (Table 6). All of these sequences shared the same codon sequence (GTT), which was also found at this position in the included TGEV sequences. The newly sequenced PRCV strains from Denmark, except for PRCV/DK/23/21.1 S, and six out of seven US PRCV sequences (not PRCV strain 1894, acc. no. OR209253.1) encoded residue I579 (Table 6), all using the same ATT codon. The included TGEV sequences encoded threonine (T) or isoleucine (I), using the codons ACT or ATT, respectively, at this position.

For the N protein, the predicted amino acid sequences from 19 out of the 20 samples were identical to those of the other, earlier, European strains, whereas the protein sequence encoded from one sample (PRCV/DK/23/12 N) was unique at two residue positions (E45 and C204). These amino acids were not found in any of the other European or US PRCV strains included in this study. Following a BLAST search of the nt sequence for PRCV/DK/23/12 N between the nt encoding residues 45 to 204, it was apparent that the amino acids at these positions were unique among the 100 most similar PRCV and TGEV sequences (National Center for Biotechnology Information (NCBI), 2024). The residues in the HOL87 sequence were identical to those of the other European strains (Table 7).

**Table 7:** Predicted amino acid residues in the PRCV N protein at positions that distinguish the US and European strains. The amino acid residues in the N protein are indicated at four positions, where the US (US column, seven strains) and the European (EU column, five strains) PRCV sequences differ, as described by Bedsted et al., 2024. The accession numbers are as follows for the US strains: DQ811787.1, KR270796.1, KY406735.1, OM830319.1, OR209252.1, OR209253.1, and OR209254.1, and for the European strains: OK078898.1, OR689863.1, OR689864.1, OM830318.1, and OM830320.1. The residue numbering is based on the sequence OR209252.1 (LEPP). X/Y indicates two different amino acids that can be found at this position. The cells that are highlighted in green indicate amino acids predicted in the newly sequenced strains from Denmark, or in HOL87 (from 1987) that are different from the other European PRCVs. Underlined amino acids are unique in this position.

### 3.6 Phylogenetic trees

**Fig. 4:**
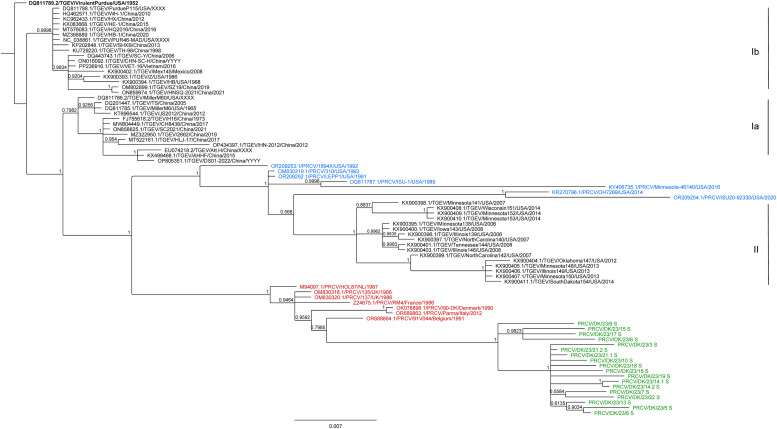
Bayesian phylogenetic tree based on partial S gene sequences of TGEVs and PRCVs. The sequences of TGEVs (black), PRCVs from Europe (red), and PRCVs from the US (blue) together with the newly sequenced PRCV strains from Denmark (green) were aligned and phylogenetic trees constructed as described in Materials and Methods. In total, 79 sequences were included. The TGEV genotypes (Ia, Ib, II) have been described by Cheng et al., 2020. The tree was rooted using the strain Virulent Purdue (acc. no DQ811789.2), highlighted in bold. Instead of collection year, "XXXX" marks lab isolates, and "YYYY" indicates that no year was registered in Genbank (National Center for Biotechnology Information (NCBI), 2024). This figure was created with Geneious Prime version 2024.0.2, and Adobe® Acrobat Pro 2024.002.20759.

**Fig. 5:**
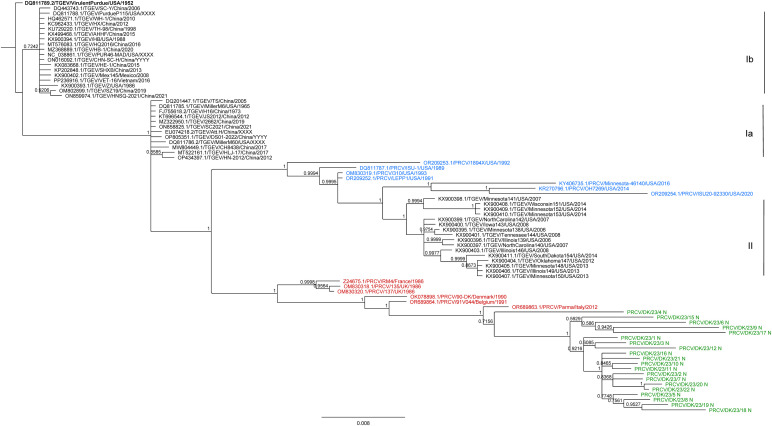
Bayesian phylogenetic tree based on the complete N gene coding sequences of TGEVs and PRCVs. The N gene sequence of TGEVs (black), PRCVs from Europe (red), and PRCVs from the US (blue), and the newly sequenced PRCV strains from Denmark from 2022 to 23 (green) were aligned and phylogenetic trees constructed as described in Materials and Methods. In total, 80 sequences were included. TGEV genotypes (Ia, Ib, II) have been described by Cheng et al., 2020. The tree was rooted using the strain Virulent Purdue (acc. no DQ811789.2), highlighted in bold. Instead of collection year, "XXXX" marks lab isolates, and "YYYY" indicates that no year was registered in Genbank (National Center for Biotechnology Information (NCBI), 2024). This figure was created with Geneious Prime version 2024.0.2, and Adobe® Acrobat Pro 2024.002.20759.

Phylogenetic trees, constructed with the MrBayes 3.2.6 algorithm, were based on partial S gene sequences (Fig. 4) and the complete N gene coding sequences (Fig. 5). In addition to the newly sequenced strains from Denmark, 14 (S) or 13 (N) PRCV sequences and 47 TGEV sequences obtained from GenBank were included (supplementary materials, Table S3) (National Center for Biotechnology Information (NCBI), 2024). Three distinct TGEV clusters were formed in the tree, corresponding to those described by Cheng et al., 2020. The US PRCV sequences were present within the genotype II cluster of TGEVs. However, the European PRCV sequences clustered separately from the TGEV sequences (as observed previously, see Bedsted et al., 2024). The strains RM4 (N) and HOL87 (S and N) as well as the newly added sequences derived from the nasal swab samples from group 1 (collected in 2022–23), also clustered with the European PRCV sequences, as expected (Fig. 4, Fig. 5). The sequences obtained from the PRCV positive samples in the screening (group 1) shared the most recent common ancestor with the strain 91V44 from Belgium, collected in 1991 (acc. no. OR689864.1) (S, Fig. 4) (96.1–97.0 % identity, supplementary materials, Table S5) or with the Parma strain from Italy, collected in 2012 (acc. no. OR689863.1) (N, Fig. 5) (97.2–98.2 % identity, supplementary materials, Table S6).

## 4 Discussion

### 4.1 Screening for respiratory pathogens

Anti-PRCV antibodies were detected in serum samples from about 90 % of the Danish pig herds tested. It should be noted that 17 of these 19 seropositive herds acquired pigs from the same vendor and the pigs could have been exposed to PRCV in the breeding herd. However, more than 80 % of the samples from the two herds that reared their own pigs (herds 8 and 17) also had anti-PRCV antibodies. Moreover, the two seronegative herds (herds 4 and 20) also bought their pigs from the same vendor as most of the other herds. This indicates that the PRCV seropositivity was not only dependent on the vendor. Herd number 4 had good quarantine conditions (separate entrance with a lobby, no air contact with other stables, and “all-in-all-out”), whereas herd number 20 had sub-optimal quarantine conditions (Nielsen et al., 2023). The lengths of the quarantine periods (10 and 13 weeks, respectively) for the two seronegative herds were among the longest among the included herds, but other herds with similar quarantine lengths also had seropositive pigs (Nielsen et al., 2023). Herd size has been associated with PRCV seropositivity in a previous study (Flori et al., 1995). The two PRCV seronegative herds tested in our study had sizes of approximately 1000–1300 sows, and were not atypical, since the other herds had a median number of sows of 1275 (Nielsen et al., 2023). Therefore, factors other than the origin of the pigs, the herd size, as well as the length and conditions of the quarantine, appear to determine whether herds had anti-PRCV seropositive pigs.

In this study, we investigated the presence of viral and bacterial respiratory pathogens in nasal swab samples from pigs in Denmark. We hypothesized that swIAV positive samples would be more likely to also test positive for other respiratory pathogens compared to swIAV negative samples. In the samples from group 1, we found that swIAV positive samples were more likely to also test positive for *A. pleuropneumoniae, M. hyopneumoniae, M. hyorhinis*, PCMV, PRCV, and SOV (Table 4). Furthermore, we found statistically significant associations between the presence of PRCV and the presence of, among others, *P. multocida*, PCMV, and SOV (Table 5). Martín-Valls et al. (2022) also analyzed the associations between different viral respiratory pathogens. Their results, from a tetrachoric correlation matrix, showed that swIAV positivity was positively correlated with the presence of PRCV, SOV, and PMCV, although this was at a farm-level and our results were based on individual (albeit pooled) samples. They also found that PRCV positivity (at a farm-level) correlated with the presence of PCV3 (not included in our analysis), PCMV, and SOV (Martín-Valls et al., 2022), which corresponds well with our results. We also found a statistically significant association between the presence of swIAV and the presence of SOV, but not PRV1, whereas another study found that swIAV was more often detected in co-infections with PRV1 compared to SOV (Graaf-Rau et al., 2023), although they did not perform statistical analyses on their data. From our analysis, we are not able to tell if, e.g. the presence of swIAV could have a confounding effect on the association between, for example PRCV and PCMV, i.e. swIAV status could have influenced both PRCV and PCMV status. Furthermore, our results could be affected by factors such as herd size and general herd health status, since, as mentioned previously, herd size has been significantly associated with PRCV seropositivity (Flori et al., 1995).

All of the most common pathogens in the swIAV negative samples, *G. parasuis, M. hyorhinis*, PCMV*, P. multocida*, and *S. suis* type 2 (Fig. 2), were also often present in swIAV positive samples in group 1. *G. parasuis, P. multocida, S. suis* (Brockmeier et al., 2002) and *M. hyorhinis* (Switzer, 1955) are considered opportunistic pathogens and are often found in healthy individuals. However, in the absence of swIAV, these bacteria may contribute to disease caused by other viruses such as PRCV or PCV2 (Brockmeier et al., 2002).

The samples from the pigs in group 2 had lower frequencies of viruses compared to the other groups, except for PCV2. We hypothesized that PRCV RNA would be detected in some of the PRCV seropositive herds. However, none of the group 2 samples, which were collected from pigs in seropositive herds, were positive for PRCV in the high-throughput RT-qPCR screening under the conditions described in Materials and Methods. Nonetheless, our results showed that in the tested herds, with and without quarantine, the gilts did not introduce PRCV into the sow herds, although the antibody responses showed that many of them had already been exposed to PRCV. This might also be related to the age of the pigs, since PRCV mostly affects younger pigs (Vangroenweghe and Thas, 2021).

Another important respiratory viral pathogen is porcine reproductive and respiratory syndrome virus (PRRSV), a notifiable agent in Denmark (‘Listebekendtgørelsen’, 2023), it was not included in the screening. However, other studies have found that PRRSV and PRCV co-infection may lead to more severe clinical signs than those seen with PRRSV or PRCV infections alone (Jung et al., 2009; van Reeth et al., 1996).

Experimental co-infection studies with PRCV and swIAV have also been performed *in vivo*. In three separate studies, pigs were inoculated with PRCV and swIAV simultaneously (Lanza et al., 1992), or with PRCV followed by swIAV some two or three (van Reeth and Pensaert, 1994), or five (Rawal et al., 2023) days later. Overall, similar clinical signs and lung lesions were seen for co-infected, as opposed to swIAV (alone)-infected, pigs in two studies (Lanza et al., 1992; Rawal et al., 2023). The third study found that co-infection gave more severe clinical signs and lung lesions than pigs infected only with swIAV, which, according to the authors, indicated that PRCV, or non-specific immunological mechanisms, also contributed to the development of clinical signs and lung lesions (van Reeth and Pensaert, 1994). Nonetheless, swIAV replication in the lung (Lanza et al., 1992; van Reeth and Pensaert, 1994), as well as the level at one-two days post-inoculation (i.e. after swIAV inoculation) (van Reeth and Pensaert, 1994; Rawal et al., 2023) and the duration (Lanza et al., 1992) of swIAV shedding (in nasal swabs), appeared to be lower in co-infected compared to pigs infected with swIAV alone.

In the screening, it could have been interesting to analyze the presence of different pathogens across ages or age groups. Unfortunately, we did not have sufficient age data registered for this purpose. Other studies have focused on the prevalence of various respiratory pathogens in pigs within different age groups (Vangroenweghe and Thas, 2021; Vereecke et al., 2023), and found, for example, across three age groups (2–5 weeks, 6–11 weeks, and 12–25 weeks) that PRCV was more prevalent in younger pigs (2–5 week old), whereas *M. hyopneumoniae* was more prevalent in fattening pigs (12–25 week old) (Vangroenweghe and Thas, 2021).

### 4.2 Characterization of PRCVs in Denmark

In a previous study, specific amino acids in the structural protein sequences were identified as being distinct between European and US PRCV strains (Bedsted et al., 2024). The predicted S and N protein sequences from the strains we have sequenced in the present study included nine and four of these residues, respectively. The residue I538 in the S protein was only found in European PRCV sequences, although more recent PRCV sequences from Europe had a valine (V), as in the US PRCV sequences (Table 6). Residue L579 in the S protein was only present in PRCV/DK/23/21.1 S, whereas the other PRCV sequences, including PRCV/DK/23/21.2 S, had I or T at this position (Table 6). This illustrates how the pattern can become less clear-cut when more sequences are included. The previous results were based on whole genome sequences of PRCV of which only five European strain sequences have been published (Bedsted et al., 2024), and it seems that these residues are not as conserved for the European PRCVs as initially suggested.

For the N protein, the sequence PRCV/DK/23/12 N was different from the others at residues number 45 and 204 (Table 7). However, it was not obvious that the sequences PRCV/DK/23/21.1 S and PRCV/DK/23/12 N differed from the others in the phylogenetic trees (Fig. 4, Fig. 5). The amino acid variations in PRCV/DK/23/21.1 S and PRCV/DK/23/12 N compared to the other newly sequenced PRCVs from Denmark were due to single nt changes, and despite these particular variations, in general, the sequences for both PRCV/DK/23/21.1 S and PRCV/DK/23/12 N were very similar to the other sequences as can be seen in the distance matrices (supplementary materials, Tables S5 and S6).

The phylogenetic trees for the S and N gene sequences closely match the trees presented in previous studies (Bedsted et al., 2024; Cheng et al., 2020), as the TGEVs grouped into three different clusters (genotypes Ia, Ib, II) (Cheng et al., 2020), with the US PRCVs clustering with TGEV genotype II, and the PRCVs from Europe, including the newly sequenced PRCVs from Denmark, clustering separately. The two strains from Europe, which were not included in a previous study (Bedsted et al., 2024), RM4 from France from 1986 (accession number Z24675.1) and HOL87 from the Netherlands from 1987 (accession number M94097.1), also clustered with the other European PRCV strains. In the phylogenetic tree based on the N gene (Fig. 5), the sequence PRCV/DK/23/4 N was located separately from the other newly sequenced PRCV strains from Denmark but it is still 96.2–97.0 % identical to these sequences, whereas the sequences of the other strains are 96.3–99.8 % identical to each other (supplementary materials, Table S6). The partial S gene sequences of the newly sequenced strains from Denmark were 97.5–99.9 % identical to each other (supplementary materials, Table S5). Had the whole S gene been sequenced instead of the fragment of 831 nt, we may have expected to see more variation at the nt level and hence lower percentage levels of identity. For example, the S gene sequences of the two PRCV strains Parma and 1/90-DK were identical in this region (supplementary materials, Table S5), although they are 98.3 % identical when comparing the whole S gene.

## 5 Conclusions

The results from the serological analyses, high-throughput RT-qPCR screening, and sequencing indicated that PRCV is circulating in pigs in Denmark. It seems that there is a higher risk of infection with other respiratory pathogens (such as swIAV, *M. hyorhinis,* PCMV, *P. multocida*, and SOV) in pigs infected with PRCV compared to uninfected pigs. This could indicate that PRCV, although not leading to severe disease by itself, may contribute to PRDC in pigs in Denmark. Therefore, testing for PRCV in pigs with respiratory disease could be relevant for improving pig health and production.

## Glossary

Gilt: Young female pig that has not farrowed

## Funding sources

This research was funded by the Danish Veterinary and Food Administration (FVST) as part of the agreement for commissioned work between the Danish Ministry of Food and Agriculture and Fisheries and the University of Copenhagen and the Statens Serum Institute. Furthermore, we received financial support from the Ole Heyes Fond. The funding sources had no involvement in the study design, in the collection, analysis and interpretation of data, in the writing and in the decision to submit the article for publication.

## Data availability

The PRCV N gene and partial S gene sequences can be found in GenBank (NCBI) under the accession numbers which can be found in the supplementary materials, Table S4.

## Ethical approval

No ethical approval was required as the screening involved standard diagnostic samples (serum or nasal swabs) collected without harm to the farm animals.

## Software

Adobe® Acrobat Pro 2024.002.20759 (www.adobe.com).

Geneious Prime version 2024.0.2 created by Biomatters. Available from https://www.geneious.com.

GraphPad Prism 10.2.2. for Windows (GraphPad Software, Boston, Massachusetts USA, www.graphpad.com).

MAFFT v7.490 Katoh & Standley 2013 (Mol Biol Evol. 30:772–780). Katoh et al. 2002 (Nucleic Acids Res. 30:3059–3066). Plugin in Geneious.

MrBayes 3.2.6. Huelsenbeck and Ronquist (2001) Bioinformatics, 17:754–755. Plugin in Geneious. The plugin in Geneious was developed by Marc Suchard and the Geneious team.

OligoAnalyzer™ Tool, IDT, Coralville, Iowa, USA. Accessed 3 June 2024. https://www.idtdna.com/SciTools.

Primer-BLAST: Ye, J., Coulouris, G., Zaretskaya, I., Cutcutache, I., Rozen, S., Madden, T.L., 2012. Primer-BLAST: a tool to design target-specific primers for polymerase chain reaction. BMC Bioinformatics. 13:134.

Standard BioTools Real-Time PCR analysis (version 1.0.2). http://standardbiotools.com.

## Author statement

We declare that this manuscript is original, has not been published before and is not currently being considered for publication elsewhere. We confirm the manuscript has been read and approved by all authors and that the order of authors listed in the manuscript has been approved by all of us. We understand that the Corresponding Author is the sole contact for the Editorial process. He is responsible for communicating with the other authors about progress, submissions of revisions and final approval of proofs.

## CRediT authorship contribution statement

**Amalie Ehlers Bedsted:** Writing – original draft, Visualization, Validation, Project administration, Methodology, Investigation, Funding acquisition, Formal analysis, Data curation. **Nicole B. Goecke:** Writing – review & editing, Methodology, Data curation. **Charlotte K. Hjulsager:** Writing – review & editing, Formal analysis, Conceptualization. **Pia Ryt-Hansen:** Writing – review & editing, Methodology, Conceptualization. **Kasama Chusang Larsen:** Writing – review & editing, Methodology. **Thomas Bruun Rasmussen:** Writing – review & editing, Supervision. **Anette Bøtner:** Writing – review & editing, Supervision, Conceptualization. **Lars E. Larsen:** Writing – review & editing, Conceptualization. **Graham J. Belsham:** Writing – review & editing, Supervision, Resources, Conceptualization.

## Declaration of competing interest

The authors declare the following financial interests/personal relationships which may be considered as potential competing interests: Amalie Ehlers Bedsted reports financial support was provided by Ole Heyes Fond. Editorial Board for Virology: Graham J. Belsham. Other authors declare that they have no known competing financial interests or personal relationships that could have appeared to influence the work reported in this paper.

## Data Availability

The PRCV N gene and partial S gene sequences can be found in GenBank (NCBI) under the accession numbers which can be found in the supplementary materials, Table S4.

## Appendix Supplementary materials

Image, application 1

Image, application 2

Image, application 3

Image, application 4
