# Supplementary material for: High-throughput screening for respiratory pathogens within pigs in Denmark; analysis of circulating porcine respiratory coronaviruses and their association with other pathogens
Source: Virus Res. 2024 Nov 26;350:199501. doi: 10.1016/j.virusres.2024.199501 (PMC11629333; doi:10.1016/j.virusres.2024.199501)
Supplement: Supplementary file 1 [file mmc1.docx]

**Table S1: Primer and probe sequences**

*Table S1. Primers and probes used in the high-throughput quantitative real-time polymerase chain reaction (RT-qPCR) screening. Modifications to the original sequence are underlined.*

| Assay name | Pathogen | Target gene | Fw primer (5′→3′) | Rv primer (5′→3′) | Probe (5′→3′) | Reference |
| --- | --- | --- | --- | --- | --- | --- |
| AP | *Actinobacillus pleuropneumoniae* | *omlA* | AGTGCTTACCGCATGTAGTGGC | TTGGTGCGGACATATCAACCTTA | FAM-CGATGAACCCGATGAGCCGCC-BHQ1 | Goecke et al. (2020a) |
| BB | *Bordetella bronchiseptica* | *dnt* | GGCGGTACTTGGGATAATAGA | GAAGAGTCCGGGGATCTTG | FAM-CGAGCATCCTGGCCGATGGGTTC-BHQ1 | Goecke et al. (2020a) |
| HP | *Glaeserella. [prev. Haemophilus] parasuis* | *ompP2* | GCTATGGTCATGAAATTTCTTTTGGTA | TACCAACACCGTATACTTTATCAAAACC | FAM-ATCGGTGATAGCATTGGTCAAGCTGG-BHQ1 | This study |
| Inf M | Influenza A | M | AGATGAGTCTTCTAACCGAGGTCG | TGCAAAGACACTTTCCAGTCTCTG | FAM-TCAGGCCCCCTCAAAGCCGA-BHQ1 | Goecke et al. (2020a) |
| Nagy2 | Influenza A | M | GGCCCCCTCAAAGCCGA | CGTCTACGYTGCAGTCC | FAM-TCACTKGGCACGGTGAGCGT-MGBEQ | Nagy et al. (2021)  *SVIP-MPv2* |
| M. hyop. | *Mycoplasma hyopneumoniae* | *mhp165* | GGCAATTCCAAGAGTTATTCAGG | TTCCGACAAGTTTTTCACCATTAG | FAM-TGATGGACTAATTGATAAAGTTCTAAACCA  TCG-BHQ1 | Goecke et al. (2020a) |
| M. hyorhi. | *Mycoplasma hyorhinis* | *p37* | CAAGCTTCYGAAACACCAAATG | CGCCAATAGCATTTGCTATATC | FAM-CAGGAGTAGTCAAGCAAGAGGATG-BHQ1 | Goecke et al. (2020a) |
| PM | *Pasteurella multocida* | *kmt1* | AGTTTGGTGTGTTGAGCCAATC | GGCAAATAACAATAAGCTGAGTAAT | FAM-TGACAACGGCGCAACTGATTGGACG-BHQ1 | This study |
| PCMV | Porcine cytomegalovirus | DPOL | CTGCCGTGTCTCCCTCTAG | ATTGTTGATAAAGTCACTCGTCTGC | FAM-CCATCACCAGCATAGGGCGGGAC-BHQ1 | Goecke et al. (2020a) |
| PCV2 | Porcine circovirus type 2 | CAP | GATGATCTACTGAGACTGTGTGA | AGAGCTTCTACAGCTGGGACA | FAM-TCAGACCCCGTTGGAATGGTACTCCTCBHQ1 | Goecke et al. (2020a) |
| Keep | Porcine respiratory coronavirus | N | ATAGACAAWCTCGCTATCGCAT | CAACCCAGACAACTCCATCT | FAM-CTAYTTAGGYACTGGACCTCATGCAG-BHQ1 | Keep et al. (2022) |
| Kim | Porcine respiratory coronavirus | N | GCAGGTAAAGGTGATGTGACAA | ACATTCAGCCAGTTGTGGGTAA | FAM-TGGCACTGCTCCCATTGGCAACGA-BHQ1 | Kim et al. (2007) |
| MV | Porcine respiratory coronavirus | N | YTCAGCCAATTTTGGTGACAGT | GATSATCCTTTGGCAAGTGG | FAM-ATGGKAGCAGTGCYAAGCATTACCC-BHQ1 | Martín-Valls et al. (2022) |
| PPIA | -^1^ | *PPIA* | CAAGACTGAGTGGTTGGATGG | TGTCCACAGTCAGCAATGGTG | FAM-CTGGTCTTGCCATTCCTGGACCCAA-BHQ1 | Starbæk et al. (2022)  *Primers (modified)*  This study  *Probe* |
| PRV1 | Porcine respirovirus 1 | F | GRRAAGACYCARAACTCAATT | CATTGACATAATCCTGGAGCA | FAM-TGAGAGCCAGTATCTGTTCACCTGC-BHQ1 | Graaf-Rau et al. (2023) |
| SOV | Swine orthopneumovirus | NP | GCCAGAGGAGTGGTTGCA | CACCACAATCTGGTGCATCA | FAM-CTGGGCTGCCTGACAATCGGAGGC-BHQ1 | Graaf-Rau et al. (2023) |
| SS | *Streptococcus suis* type 2 | *gdh* | CCAAAGCTTCATGACTGAATTGC | CGACCACCGACACCGATG | FAM-ACACATCGGACCTTCACTTGACGTC-BHQ1 | Goecke et al. (2020a) |

*^1^The PPIA assay, which targets the porcine housekeeping gene peptidyl prolyl isomerase A (PPIA), was included as a positive control.*
