# Supplementary material for: High-throughput screening for respiratory pathogens within pigs in Denmark; analysis of circulating porcine respiratory coronaviruses and their association with other pathogens
Source: Virus Res. 2024 Nov 26;350:199501. doi: 10.1016/j.virusres.2024.199501 (PMC11629333; doi:10.1016/j.virusres.2024.199501)
Supplement: Supplementary file 2 [file mmc2.docx]

**Table S2: Primers for PRCV sequencing**

*Table S2: Primers designed for cDNA synthesis, amplification by PCR, and sequencing of the PRCV partial S gene and the N gene.*

| Name | Sequence (5′→3′) |
| --- | --- |
| 22283R | TGTTACACCTAGGGTTGCCA |
| 22437R | AATCTTCATCAACTGTACCTAAACCAG |
| PRCVseqSF | TTACTGCTAATTTGAATAATGGATT |
| PRCVseqSR | TATGTTAGAATAGGTTATGACAGG |
| PRCVseqNF1 | GAGTGAGCAAGAAAAATTATTACA |
| PRCVseqNR1 | TTAGAGTCACTACGTTCTTTAGA |
| PRCVseqNR2 | CCAATTAGTAGTAAGGTTAAAACTG |
